# Individuals with type 2 diabetes have higher density of small intestinal neurotensin-expressing cells

**DOI:** 10.1007/s11010-023-04698-z

**Published:** 2023-03-15

**Authors:** Filipa P. Ferreira, Sofia S. Pereira, Madalena M. Costa, Marta Guimarães, Nicolai J. Wewer Albrechtsen, Jens J. Holst, Mário Nora, Mariana P. Monteiro

**Affiliations:** 1 Department of Anatomy, UMIB–Unidade Multidisciplinar de Investigação Biomédica, Instituto de Ciências Biomédicas Abel Salazar (ICBAS), University of Porto, Rua Jorge Viterbo Ferreira 228, Building 1.3, 4050-313 Porto, Portugal; 2 ITR–Laboratory for Integrative and Translational Research in Population Health, Porto, Portugal; 3 Department of General Surgery, Centro Hospitalar de Entre Douro E Vouga, Santa Maria da Feira, Portugal; 4 Department of Biomedical Sciences, Faculty of Health and Medical Sciences, University of Copenhagen, Copenhagen, Denmark; 5 Faculty of Health and Medical Sciences, Novo Nordisk Foundation Center for Basic Metabolic Research, University of Copenhagen, 2100 Copenhagen, Denmark; 6 Department of Clinical Biochemistry, Rigshospitalet, Copenhagen, Denmark

**Keywords:** Neurotensin, Enteroendocrine cells, Small intestine, Obesity, Type 2 diabetes, Bariatric surgery

## Abstract

Neurotensin (NT) is a gastro-intestinal hormone involved in several pathways that regulate energy and glucose homeostasis. NT was hypothesized to act in synergy with incretin hormones to potentiate its anti-diabetic effects. Additionally, circulating NT levels were shown to rise after bariatric surgery-induced weight loss. Knowledge of NT-secreting cells distribution along the small intestine and its variation according to diabetes status could provide insights on NT role in mediating type 2 diabetes (T2D) improvement after bariatric surgery. So, our aims were to characterize NT-expressing cell distribution along the human small intestine and to compare the relative density of NT-expressing cells in the small intestine of individuals with and without T2D undergoing bariatric surgery for obesity treatment. Autopsy-derived small intestine fragments (*n* = 30) were obtained at every 20 cm along the entire intestinal length. Additionally, jejunum biopsies (*n* = 29) were obtained during elective gastric bypass interventions from patients with (*n* = 10) or without T2D (*n* = 18). NT-expressing cells were identified by immunohistochemistry and quantified via computerized morphometric analysis. NT-expressing cell density increased along the human small intestine. NT-expressing cell density was significantly higher from 200 cm distal to the duodenojejunal flexure onward, as well as in subjects with T2D when compared to those without T2D. NT-expressing cell density increases along the human small gut, and a higher density is found in individuals with T2D. This finding suggests a potential role for NT in the mechanisms of disease and T2D improvement observed after bariatric surgery.

**Supplementary Information:**

The online version contains supplementary material available at 10.1007/s11010-023-04698-z.

## Introduction

Bariatric surgery is the most effective long-term treatment for severe obesity [1]. In addition, the majority of patients submitted to bariatric surgery also experience significant improvements or even resolution of obesity-associated comorbidities, such as type 2 diabetes (T2D) [2, 3, 4]. The anti-diabetic effect of bariatric surgery has been attributed to modification of endocrine dynamics derived from the anatomical rearrangement of the gastrointestinal (GI) tract, in addition to the decreased caloric uptake and weight loss [1, 5].

However, the impact of bariatric surgery procedures on GI hormone secretion, with a well-established role on energy and glucose metabolism control, such as GLP-1, GIP and PYY, was shown to diverge according to the anatomical rearrangement derived from the technical procedure performed. Neurotensin (NT) is a less well-characterized GI hormone, predominantly secreted at the small intestine [6] but also at the nervous system [7, 8]. NT effects on the central nervous system have been more extensively characterized and include regulation of dopaminergic, ghrelin and leptin-associated pathways that mediate satiety and food intake [9, 10, 11, 12]. At the small intestine, NT secretion and release occurs predominantly in response to fat intake [13] and has been hypothesized to promote fat absorption through entero-hepatic cycle bile acid regulation [14] and gastric emptying [15]. Additionally, there is accumulating evidence that NT plays a role within the GI hormone network by acting synergistically with GLP-1 and PYY on delaying gastric emptying and inhibiting food intake [16] through mechanisms that remain elusive [17]. Moreover, NT’s role in glucose homeostasis seems to be highly complex, as it was shown to stimulate insulin secretion at low glucose levels, while inhibiting insulin release in the presence of high glucose levels [18, 19]. Furthermore, an in vitro study demonstrated that NT protects pancreatic β-cells against apoptosis [20]. Overall, the aforementioned evidence on NT leads to the hypothesis that this hormone could act as an incretin hormone, alongside GIP and GLP-1 [17, 21].

Considering that neuroendocrine cell density varies throughout the small intestine [22], we hypothesize that the NT-secreting cells distribution in the human small intestine varies in different intestinal length intervals: 0–80, 81–200 and 201–700 cm, that are equivalent to intestinal edge of a biliopancreatic limb with 80 cm in the standard/classic Roux-en-Y gastric bypass (RYGB) or 200 cm in the long biliopancreatic limb bypass and so to have an impact on patients systemic metabolic status and RYGB clinical outcomes.

## Materials and methods

### Patient selection and histologic procedures

#### Cadaveric small intestine fragments

Small intestines from 30 adult human cadavers deceased from accidental causes were harvested. Only cadavers without macroscopic evidence of hepatic, pancreatic, intestinal, or neoplastic disease, previous abdominal surgery and visible signs of putrefaction were included in this study. The group’s demographic and anthropometric characteristics are depicted in Table 1.

**Table 1:** Demographic and anthropometric characteristics of human cadavers

Cadaver (*n*)	30
Collected fragments per cadaver (*n*)	27.4 ± 0.9
Age (years)	65 ± 3.5
Ratio male:female	17:13
Weight (kg)	67.5 ± 2.1
BMI (kg/m^2^)	25.8 ± 0.7
Male	26 ± 1.3
Female	25.5 ± 0.7
BMI category (*n*, %)	
Normal weight	12 (40%)
Overweight	15 (50%)
Obesity	3 (10%)
Intestinal length (cm)	533.4 ± 18.1

Data are presented as mean ± standard error or number (%), as appropriate. BMI, body mass index

The small intestine was detached from the mesentery from the duodenojejunal flexure until the ileocecal valve. The small intestinal length was then measured, and 1-cm-wide sections comprising the full thickness of the small intestine were systematically collected at every 20-cm interval. Tissue fragments were fixed in 4% buffered formaldehyde for 24 h before being subjected to routine automatic tissue-processing procedures for light microscopy. After the identification of small intestinal mucosa in hematoxylin–eosin-stained slides, tissue microarrays (TMA) paraffin blocks were mounted containing sequential 2-mm tissue cores representing every single intestinal mucosa sample from each cadaver. Liver and pancreatic tissue fragments were included in each block as negative and positive controls, respectively. Tissue section (3 µm) were mounted on Superfrost (Thermo Scientific, Waltham, MA) slides.

#### Small intestine surgical biopsies

Small intestine fragments were harvested by transversely sectioning the intestinal edge created to perform the gastro-enteric anastomosis construction as standard procedure, during elective gastric bypass surgeries. Tissue fragments were collected from the small intestine located at 60–90 cm (*n* = 28) from the duodenojejunal flexure of patients with obesity and concomitant T2D (*n* = 10) or without T2D (*n* = 18). The subjects anthropometric and demographic features are depicted in Table 2.

**Table 2:** Demographic and anthropometric characteristics of subjects submitted to bariatric surgery

	Non-T2D subjects (*n* = 18)	T2D subjects (*n* = 10)	*p* value
Age (years)	41.33 ± 2.21	51.20 ± 2.27	< 0.001
Ratio male:female	4:14	3:7	0.674
BMI (kg/m^2^)	39.32 ± 1.03	40.70 ± 1.57	0.621
Distance from Treitz ligament (cm)	77.78 ± 3.81	75.00 ± 5.00	0.664
Fasting glucose (mg/dL)	96.89 ± 1.72	169.90 ± 22.10	0.001
HbA_1c_ (%)	5.23 ± 0.15	6.84 ± 0.38	< 0.001
Insulin (pq/ml)	16.64 ± 3.52	18.15 ± 3.38	0.781
HOMA-IR	4.13 ± 1.01	7.52 ± 1.53	0.066
HOMA-β	187.35 ± 35.95	82.37 ± 19.97	< 0.001

Results are presented as mean ± standard error. BMI, body mass index; HbA1c, hemoglobin A1c; HOMA-B, homeostasis model assessment of β-cell function; HOMA-IR, HOMA of insulin resistance; T2D, type 2 diabetes

Immediately after surgical harvest, small intestinal tissue biopsies were immersed in 4% buffered formaldehyde, preserved for 72 h and then routinely processed for paraffin embedding and optical microscopy. Tissue sections (3 µm) were mounted on Starfrost (Knittel Glass, Germany) slides.

### Immunohistochemistry techniques

NT-expressing cell detection was performed using an anti-NT specific antibody (3488-7), kindly provided by Nicolai J. Wewer Albrechtsen and Jens J. Holst, from the University of Copenhagen. Commercial antibodies were used to identify neuroendocrine cell (anti-chromogranin-A antibody, ab17064, Abcam, Cambridge, UK), GLP-1 (ab22625, Abcam) and GIP (ab30679, Abcam)-producing cells.

Antigen retrieval was performed in the microwave using 10 mM citrate buffer (pH 6.0). Endogenous peroxidase was blocked with 3% H_2_O_2_ for 20 min, followed by incubation with normal serum for 30 min. Incubation with primary antibodies (Anti-chromogranin-A 1:200; Anti-NT 1:5000 Anti-GIP 1:500 and Anti- GLP-1 1:4000 in 5% BSA) was performed overnight at 4ºC. Incubation with secondary biotinylated polyclonal antibodies (1:200, EO35301-2 or EO35401-2, Dako, Glostrup, Denmark) was performed for 30 min, followed by the application of avidin–biotin complex (ABC) (1:100 dilution in 5% BSA; Vector Laboratories, Peterborough, UK) for 30 min. Diaminobenzidine was the chosen chromogen (3,3’-Diaminobenzidine, Dako), and the revelation lasted 2 min for chromogranin-A, 30 s for GIP and GLP-1 and 10 s for NT. All sections were counterstained with Mayer’s hematoxylin (HX390929, Merck, Darmstadt, Germany).

### Data retrieval and analysis

Immunohistochemistry-stained slides were scanned using a slide scanning system (Olympus VS110), and images were acquired through the VS-ASW software (version 2.3 for Windows, Olympus, Tokyo, Japan). Tissue fragments with microscopic signs of autolysis were excluded from analysis. Images were analyzed with the aid of an image processing software (ImageJ, National Institutes of Health) with a color deconvolution plugin that separates the stained area from the initial image allowing quantification of the percentage area specifically stained by NT or chromogranin A antibody. The percentage of stained area (%SA) within the small intestine mucosa total area for each given molecular marker was quantified, and the ratio of the %SA for NT/chromogranin A was calculated as a surrogate of the relative proportion of NT-expressing cells among the intestinal neuroendocrine cell population. GIP and GLP-1 expressing cells were also quantified in the small intestine surgical biopsies using a similar analysis method as previously described [23].

The cadaveric data were aggregated according to the intestinal length at which tissue fragments were collected in accordance with metrics commonly used in gastric bypass surgeries for the construction of biliopancreatic limb: 0–80, 81–200 and 201–700 cm, thus equivalent to the intestinal edge of a biliopancreatic limb with 80 cm in the standard/classic RYB or 200 cm in the long biliopancreatic limb bypass, also denominated “metabolic gastric bypass,” after demonstrating having additional anti-diabetic effects as compared to the classical intervention [24].

### Statistical analysis

Statistical analysis was performed using the GraphPad Prism version 8.0.1 for Windows (GraphPad Software, La Jolla California USA). Results are expressed as mean ± standard error of the mean (mean ± SEM) unless otherwise specified. Statistical significance was considered at a p value of < 0.05. A *t*-test or a Mann–Whitney test was used to compare two groups, depending on the variables’ distribution. In order to compare three or more groups, a one-way ANOVA or a Kruskal–Wallis test was performed.

## Results

### NT-expressing cell density increases along the human small intestine

**Fig. 1:**
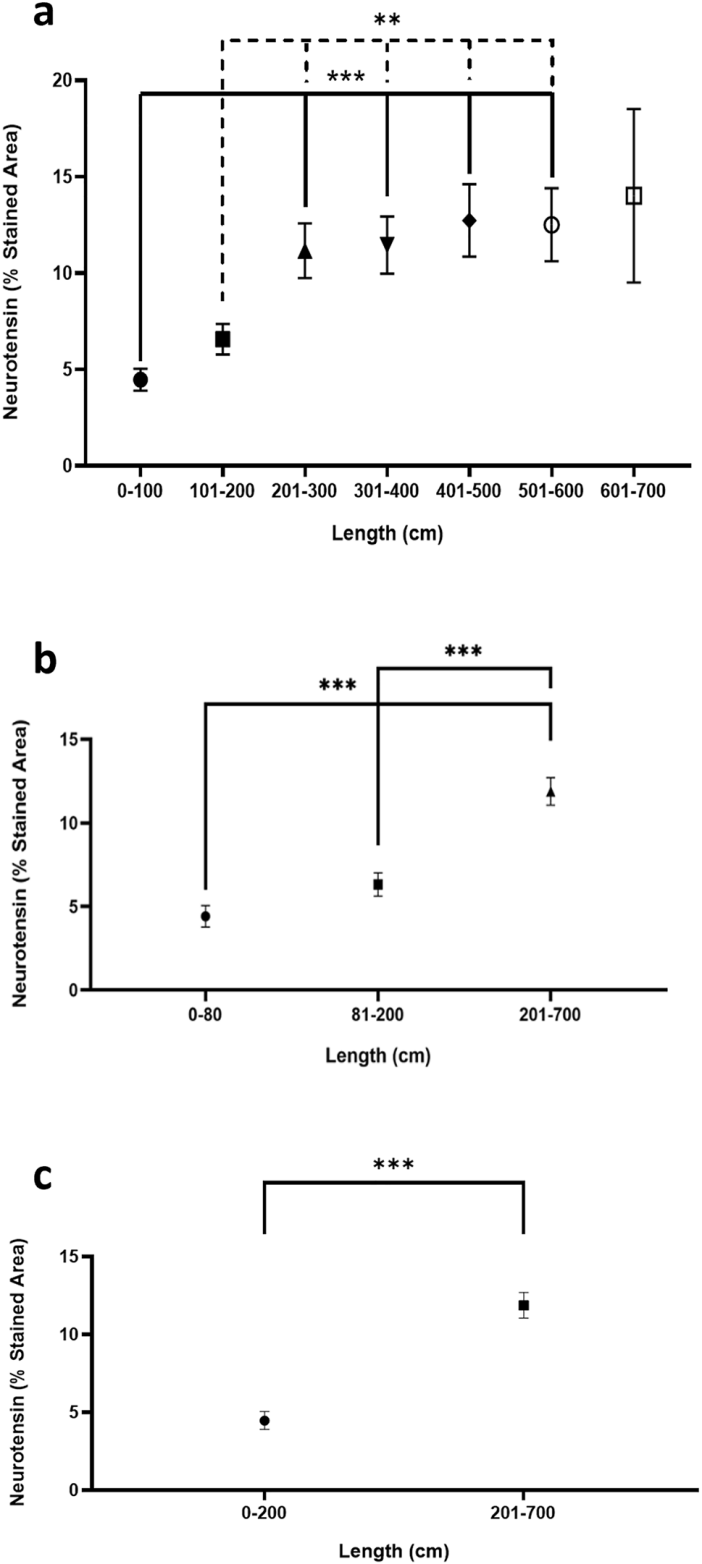
Percentage of neurotensin-stained area at 100-cm intervals (**a**). Grouped data at 0–80-cm, 81–200-cm and 201–700-cm intervals (**b**). Grouped data from 0–200-cm to 201–700-cm intervals (**c**). Statistical analysis: *p < 0.05; **p < 0.01; ***p < 0.001

The percentage of NT-stained area was significantly higher on small intestine fragments located at 200 cm from the duodenojejunal flexure, reaching a plateau across the remaining intestinal length (Fig. 1A).

The 201–700-cm interval, which corresponds to the intestinal segment that will be used for the surgical construction of the alimentary and common limbs in the metabolic gastric bypass, presented a significantly higher percentage of NT-stained area when compared to the 0–80 cm (11.87 ± 0.83% vs 4.41 ± 0.64%; *p* < 0.001) and 81–200 cm (11.87 ± 0.82% vs 6.32 ± 0.70%; *p* < 0.001) intervals (Fig. 1B). Although there was no statistical difference between the 0–80 and 81–200-cm intervals (Fig. 1B), a significantly higher percentage of NT-stained area was identified at distal (200–700 cm) small gut when compared to proximal (0–200 cm) gut (11.87 ± 0.82% vs 4.47 ± 0.57%; *p* < 0.001) (Fig. 1C). The differences are similar when we compare the genders in separate (male: 10.71 ± 1.85 at 200–700 cm vs 4.89 ± 1.06 at 0–200 cm; female: 15.09 ± 3.19 at 200–700 cm vs 4.79 ± 1.23 at 0–200 cm) (Online Resource 1).

### NT-expressing cell density is higher in subjects with T2D

Patient baseline clinical features were not significantly different when subjects with and without T2D were compared, except for age, fasting glucose, HbA1c and HOMA-B, which as expected were significantly higher in individuals diagnosed with T2D.

**Fig. 2:**
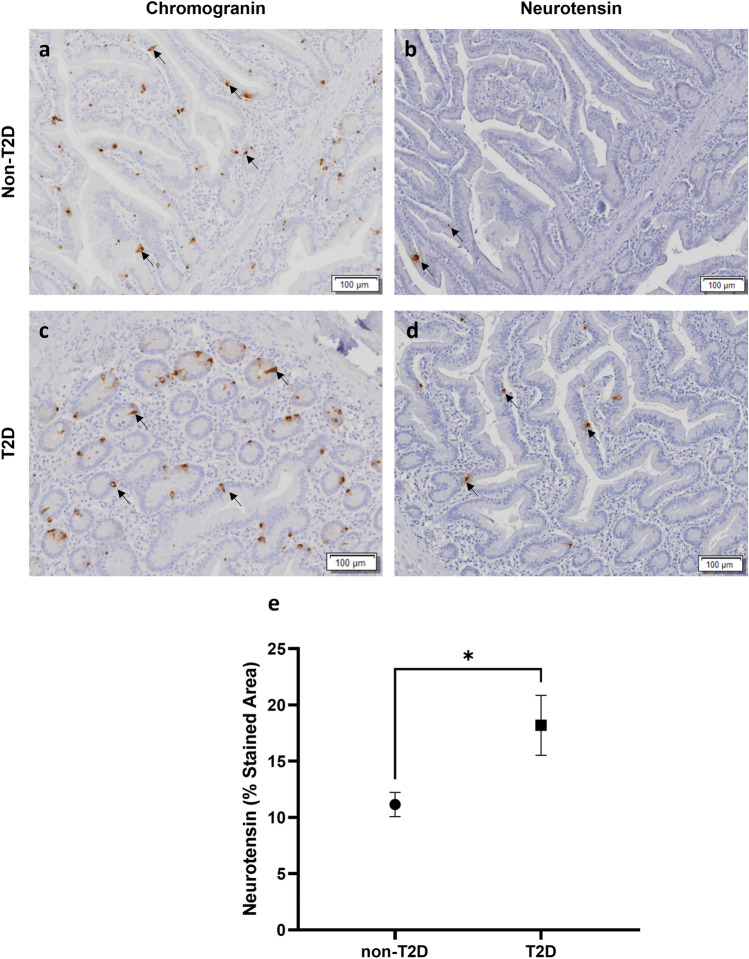
Small intestinal mucosal biopsy from a patient without type 2 diabetes (T2D) stained for chromogranin-A (**a**) and for neurotensin (**b**). Small intestinal mucosal biopsy from a patient with T2D stained for chromogranin-A (**c**) and for neurotensin (**d**). Arrows indicate some stained cells. Comparison of the stained area for neurotensin in the small intestine of subjects with obesity without and with concomitant T2D (**e**). Statistical analysis: *p < 0.05

**Fig. 3:**
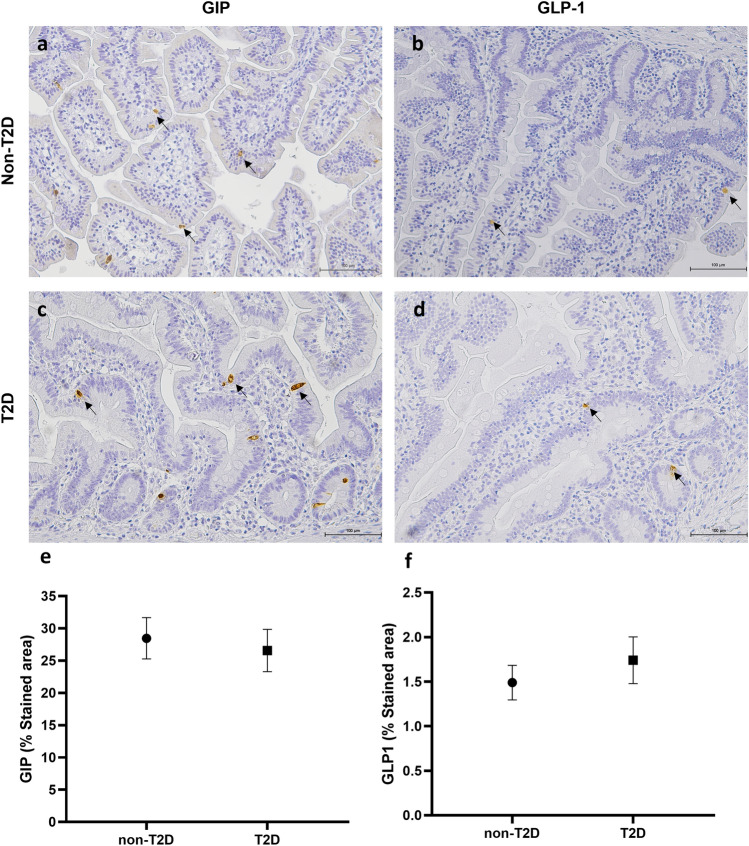
Small intestinal mucosal biopsy from a patient without type 2 diabetes (T2D) stained for GIP (**a**) and for GLP-1 (**b**). Small intestinal mucosal biopsy from a patient with T2D stained for GIP (**c**) and for GLP-1 (**d**). Arrows indicate some stained cells. Comparison of the stained area for GIP (**e**) or GLP-1 (**f**) in the small intestine of subjects with obesity without and with concomitant T2D

Subjects with T2D had a significantly higher of NT-expressing cells density in the small gut when compared to that of individuals without T2D (18.19 ± 1.075% vs 11.16 ± 1.075; *P* < 0.05) (Figs. 2). No differences were observed for GIP and GLP-1 when compared patients with or without T2D (Fig. 3).

## Discussion

The demonstration that circulating NT levels are higher in patients submitted to RYGB with a long biliopancreatic limb gastric bypass as compared to those that underwent a standard procedure [25], has raised our interest on exploring NT cells distribution along the human small gut, in order to gain further insight into its potential contribution for the metabolic outcomes achieved by different anatomical modifications of the GI tract. Previous studies that aimed to assess the intestinal NT-producing cell distribution were either conducted on animals models [6] or were limited to few anatomical segments [26, 27, 28, 29] of the human small intestine. Therefore, it was our goal to contribute for filling the lack of knowledge by performing a thorough characterization of NT-secreting cells along the human small intestine.

This study allowed to demonstrate that there are significant differences of NT-expressing cells relative density along the small intestine, which is higher distally to the first 100 cm onward as compared to the initial segment. Additionally, a higher percentage of NT-positive cells is fount at the 201–700-cm interval as compared to the 0–80 and 81–200-cm intervals, which corresponds to the intestinal segment used to create the alimentary and common limb in a long biliopancreatic limb gastric bypass.

These results are consistent with a previous report in which described the presence of a higher percentage of NT-positive cells in distal as compared to proximal small intestine [30]. Therefore, a long biliopancreatic limb gastric bypass surgery not only shortens the absorptive intestinal length, but also accelerates the contact of more distal intestinal mucosa with undigested nutrients, which could potentially lead to greater stimulation of NT secreting cells, as demonstrated in animal models [31]. This stimulatory effects would then be key to upregulate NT-modulated pathways, such as the putative enhancement of the incretin effect [32], and consequently the anti-diabetic effects observed after gastric bypass.

Moreover, it was our aim to investigate whether the density of NT-secreting cells in the small intestine of patients with T2D present was significantly different from those of subjects without this disease. Our work further demonstrates that individuals with T2D have a higher density of NT-positive cells in the proximal small intestine than observed in unaffected individuals. Despite the fact that no significant differences were previously observed of *NT* gene expression in the small intestine of patients with T2D as compared to those without this disease [33], our observation is consistent with the finding that higher levels of pro-NT, a stable precursor of NT, predict the development of T2D in adults [34]. Similarly, higher pro-NT levels in children were found to be associated with impaired β-cell function and weight gain later in life [35].

In addition, Pro-NT levels increase after gastric bypass [36] and are positively associated with weight loss and improved insulin sensitivity observed after metabolic surgery [37]. Indeed, NT’s effects appear to be modulated by nutritional status, through mechanisms that are currently unknown, although recently have been hypothesized to be linked to biliary acid metabolism and mediated via to farnesoid X receptor (FXR) activation [38]. Moreover, enhanced incretin effect has been consistently reported after gastric bypass surgery [39, 40], an effect that has also been suggested to depend on bile acid FXR activation [41, 42]. FXR has been shown to contribute to an enhanced incretin effect by mediating the epigenetic modulation of β-cell GLP-1 receptor expression in mice [43].

Thus, we hypothesize that the nutritional status and NT stimulation differentially impact on its downstream effects. According to this hypothesis, in the context of a fat-rich diet, nutrients would not only stimulate NT secretion but also induce its inhibitory action on FXR, consequently inhibiting GLP-1 secretion. However, after gastric bypass an earlier stimulation of the distal small intestine with higher density of NT cells, in a less nutrient-rich environment, would induce NT-mediated FXR activation and increase the incretin action.

Although no direct comparisons of the NT-expressing cells’ relative density in cadaveric biopsies and surgical biopsies were made, given the natural differences between these tissues, our data have shown that NT-expressing cell density is higher in patients with obesity obtained from surgical biopsies than in the cadaveric intestinal fragments obtained from normal weight subjects. This is in line with previous reports and could reflect an adaptive response to increased dietary fat intake [35].

As far as we know, this is the first systematic evaluation of NT cell distribution in human tissue fragments collected at regular intervals along the entire length of the human small in which a computerized morphometric analysis method was applied to quantify stained cells in a non-subjective way. In addition, there are no previous studies comparing the NT intestinal protein expression between patients with and without T2D, complementing the previous studies that evaluated *NT* gene expression and pro-NT plasmatic levels [33, 34].

Our herein data, showing that NT-expressing cell density increases along the human small intestine, firstly provide a morphological support for the finding that individuals with obesity submitted to a 200-cm-long biliopancreatic limb RYGB have higher NT-circulating levels [25]. We have also shown that individuals with T2D have a higher NT-expressing cell density in the same intestinal location; thus, we could hypothesize that a greater stimulation of NT cell would occur, providing an explanation for the additional metabolic benefits of the 200-cm-long biliopancreatic limb RYGB in this patient population [24]. Overall, our data provide further evidence in support of performing a 200-cm-long biliopancreatic limb RYGB in individuals with obesity and type 2 diabetes, with the rationale of increasing NT-expressing cells stimulation aimed at maximizing the outcomes of bariatric surgery.

This study presents some limitations that need to be acknowledged, namely the fact that only a small number of patients with T2D were included and the quantification of the relative cell density was performed at a single segment of the small intestine. Further studies should aim to compare NT-secreting cell patterns across different intestinal segments across the length of the small gut in a larger cohort of patients with and without concomitant T2D. In addition, we do not provide any data on morphological vs functional correlations, since we do not have plasmatic samples at the type of the surgery to assess circulating NT dynamics and so to perform a mechanistic physiological study.

In conclusion, the relative density of the NT-producing cells is not uniform across the human small intestine and seems to vary according to T2D status. These data may suggest the role of the differential stimulation of intestinal NT cell populations for the NT secretion dynamics and its potential impact on T2D.

### Supplementary Information

Below is the link to the electronic supplementary material.

Supplementary file1 (PDF 202 KB)

## Data Availability

The datasets generated during and/or analyzed during the current study are not publicly available due to include patients’ confidential data but are available from the corresponding author on reasonable request.
