# Supplementary material for: Individuals with type 2 diabetes have higher density of small intestinal neurotensin-expressing cells
Source: Mol Cell Biochem. 2023 Mar 15;478(12):2779–87. doi: 10.1007/s11010-023-04698-z (PMC10627918; doi:10.1007/s11010-023-04698-z)
Supplement: Supplementary file 1 — Supplementary file1 (PDF 202 KB) [file 11010_2023_4698_MOESM1_ESM.pdf]

Individuals with type 2 diabetes have higher density of small intestinal neurotensin expressing cells

Filipa P. Ferreira[1,2], Sofia S. Pereira[1,2], Madalena M. Costa[1,2], Marta Guimarães[1,2,3], Nicolai J. Wewer Albrechtsen[4,5,6], Jens J. Holst[4,5], Mário Nora[3], Mariana P. Monteiro[1,2]

[1]UMIB - Unidade Multidisciplinar de Investigação Biomédica, ICBAS - Instituto de Ciências Biomédicas Abel Salazar, Universidade do Porto, Porto, Portugal

[2]ITR - Laboratory for Integrative and Translational Research in Population Health, Porto, Portugal

[3]Department of General Surgery, Centro Hospitalar de Entre o Douro e Vouga, Santa Maria da Feira, Portugal

[4]Department of Biomedical Sciences, Faculty of Health and Medical Sciences, University of Copenhagen, Copenhagen, Denmark

[5]Novo Nordisk Foundation Center for Basic Metabolic Research, Faculty of Health and Medical Sciences, University of Copenhagen, 2100 Copenhagen, Denmark

[6]Department of Clinical Biochemistry, Rigshospitalet, Copenhagen, Denmark

Corresponding Author: Sofia S. Pereira, sspereira@icbas.up.pt

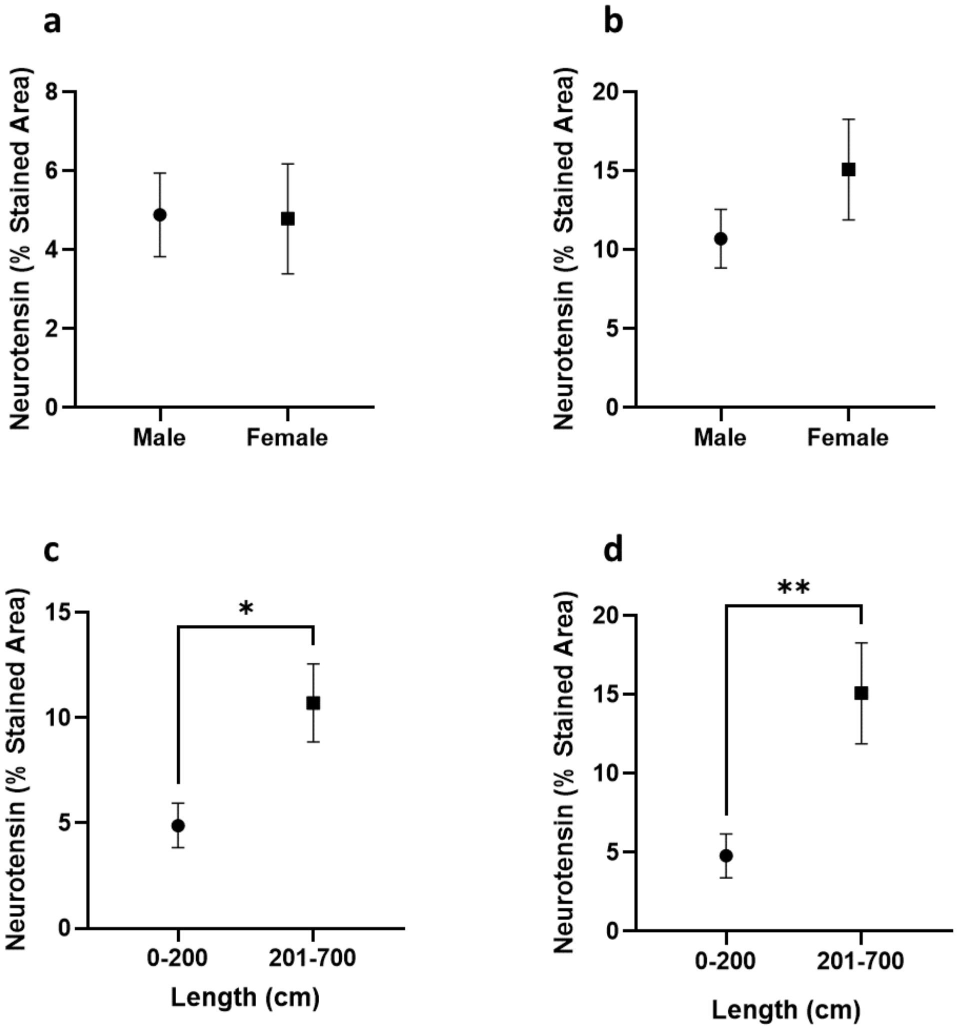

Online Resource 1 - Percentage of Neurotensin-stained area at 0-200 cm (a) and 201-700cm (b) small intestinal intervals comparing males and females' subjects. Grouped data from 0-200cm and 201-700cm intervals, in males (c) and females (d), in separate. Statistical analysis: * p<0.05; ** p<0.01.
